# Effectiveness of ^18^F-FDG PET/CT in the diagnosis and staging of osteosarcoma: a meta-analysis of 26 studies

**DOI:** 10.1186/s12885-019-5488-5

**Published:** 2019-04-05

**Authors:** Fanxiao Liu, Qingyu Zhang, Dongsheng Zhou, Jinlei Dong

**Affiliations:** 1 Department of Orthopaedics, Shandong Provincial Hospital affiliated to Shandong University, No.324, Road Jing Wu Wei Qi, Jinan, 250021 Shandong China; 2 Department of Orthopaedic Surgery, Physical Medicine and Rehabilitation, University Hospital of Munich (LMU), Campus Grosshadern, Marchioninistrasse 23, 81377 Munich, Germany; 3 Department of Orthopeadics, Qilu Hospital, Shandong University, Jinan, Shandong China

**Keywords:** ^18^F-FDG PET, PET/CT, Metastases, Meta-analysis, Osteosarcoma, Diagnostic accuracy

## Abstract

**Background:**

Multiple trials have attempted to assess the diagnostic value of ^18^F-fluorodeoxyglucose positron emission tomography/computed tomography (^18^F-FDG PET/CT) in osteosarcoma with results remaining inconclusive. This study aims to investigate the effectiveness of ^18^F-FDG PET and PET/CT in the diagnosis, staging, recurrence and metastasis formation observations of osteosarcoma through systematic review followed by meta-analysis.

**Methods:**

Three electronic databases, Medline/PubMed, Embase and the Cochrane Library were utilized in this study. Eligible studies that assessed the performance of ^18^F-FDG PET/CT for the diagnosis, staging, restaging and recurrence monitoring of osteosarcoma were retrieved utilizing specific search criteria. After screening and diluting out the non-conforming articles, all relevant articles and their data were identified and extracted to calculate the summary metrics involving sensitivity, specificity, diagnostic odd ratio (DOR), and area under the curve (AUC) to determine the effectiveness of ^18^F-FDG PET in diagnosing osteosarcoma clinically.

**Results:**

Out of 1976 articles searched, twenty-six studies were identified that were viable. All data from these articles, utilized in the quantitative analyses, showed after meta-analysis that when utilizing ^18^F-FDG PET or PET/CT it was better with a success rate of 90–100% for detecting primary lesions and distant metastases of patients with osteosarcoma. Similar results were also obtained for detecting lung and bone metastases in a subgroup analysis.

**Conclusions:**

As such the investigation demonstrated that ^18^F-FDG PET and PET/CT are very accurate for the diagnosis, staging and recurrence monitoring of osteosarcoma. ^18^F-FDG-avid lesions should be further examined in osteosarcoma, especially for suspicious lung lesions.

## Background

Osteosarcoma is the most frequent type of primary bone malignancy in childhood and adolescence, which originates from primitive mesenchymal stem cells that improperly form osteoblasts that then deposit malignant osteoid [1]. The combination of high-dose chemotherapy and limb-salvage surgery has been shown to prolong the overall survival of localized osteosarcoma to 65~70% [2]. However, the prognosis of patients with radiographically discernable distant metastases is still unfavorable due to a large majority of occult metastases present in lung, and the minority in bone, lymph node and other parts of the body [3]. Moreover, after limb-salvage operations, approximately 13.5% of patients have a local recurrence of the sarcoma [4]. The outcome of local recurrent osteosarcoma is even worse than for patients with metastases alone [5]. Therefore, accuracy and early detection of local recurrence and distant metastases formation have a crucial role in the risk stratification and the treatment of osteosarcoma.

Several traditional imaging modalities have been used for the diagnosis, staging and treatment monitoring of osteosarcoma, such as plain radiographs, computed tomography (CT) and magnetic resonance imaging (MRI). ^18^F-fluorodeoxyglucosepositron emission tomography (^18^F-FDG PET), through detecting the high uptake of ^18^F-FDG, a radioactive analogue of glucose, could identify sites with increased metabolic activity of various malignant tumors. More recently, PET/CT, which combines metabolic data from PET and imaging data from conventional CT, seems to be far more reliable in diagnosing malignancies.

A previous clinical study [6] had demonstrated that osteosarcoma was ^18^F-FDG-avid. Uptake of ^18^F-FDG was applied for the diagnosis, chemotherapy response assessment, prognosis prediction and guidance of biopsies of osteosarcoma [7]. Compared with bone scintigraphy, ^18^F-FDG PET and PET/CT could identify bone, lung and other metastatic lesions. Subsequently, ^18^F-FDG PET and PET/CT have an advantage in assessing local recurrence as they are not affected by imaging artifacts.

Multiple studies have attempted to assess the diagnostic accuracy of ^18^F-FDG PET and PET/CT in osteosarcoma. However, there seems to be considerable methodological variability, including the methods for evaluating the FDG uptake and the standardized uptake value (SUV) at determining whether the lesions are positive. Results remain inconclusive. Recently, two systematic review coupled meta-analyses [8–10] tried to further clarify this issue, but none of the included studies specifically aimed at osteosarcoma and did not statistically analyze the retrieved data. ^18^F-FDG PET or PET/CT have not been considered as the standardized components of the diagnostic algorithm for osteosarcoma. To reach a more precise result on this topic, the present study sought to systematically collected previously published data from literatures and performed a statistically evaluation using meta-analysis to see if ^18^F-FDG PET/CT is far more efficient at diagnosing osteosarcoma then present detection paradigms.

## Methods

### Search strategy, inclusion and exclusion criteria

Two investigators, blinded, independently and repeatedly, performed a systematic computerized article search using three databases (Medline/PubMed, Embase and the Cochrane Library) with combinations of following key words: “positron emission tomography” [all field] OR “PET” [all field] AND “osteosarcoma” [all field] OR “bone sarcoma” [all field]. No language limitations were imposed. This search process was completed on March 1, 2018 with no language and search limitations. Additionally, bibliographies of included studies were also searched by hand to explore any potentially eligible trials.

The targeted studies in the meta-analysis had to fulfill all following criteria: a) clinical trials assessing the usefulness of ^18^F-FDG PET or PET/CT in the diagnosis, staging, restaging and recurrence monitoring of osteosarcoma; b) patients with osteosarcoma clinically diagnosed by histopathology, follow-up or other reference methods; c) sufficient data were provided to calculate the number of true-positive (TP), false-positive (FP), false-negative (FN) and true negative (TN) cases; d) if more than one article contained overlapping data, the most comprehensive or recent article was included; and e) ^18^F-FDG was intravenously administered as an inducer.

Exclusion criteria for this meta-analysis were: a) in vitro or animal studies; b) trials with fewer than five participants with osteosarcoma; c) posters presented at conferences/congresses (due to the lack of data and methodology description); d) not original research (reviews, editorials, meta-analyses, letters and comments).

### Data extraction and quality assessment

The following main information were extracted from original articles: first author’s surname, year of publication, source of studies, basic characteristics of the participants (numbers, age and gender), study design, inclusion interval, technical details (image devices, injection dose and methods of image analysis) and the time between injection and image acquisition). Additionally, the cases of TP, FP, TN and FN were extracted directly or recalculated through data presented in original articles based on different lesions such as primary, recurrence and metastases of lung and bone.

The methodological quality of included studies was appraised utilizing the QUADAS tool [11], which is composed of 14 items. Study following at least nine items of these scores was deemed as high quality and was included in this investigation.

### Statistical analysis

This systematic review and meta-analysis confirmed the standardized items described by “the Preferred Reporting Items for Systematic Reviews and Meta-Analyses (PRISMA)” statement [12]. To assess the accuracy of ^18^F-FDG PET and PET/CT on the diagnosis, staging and recurrence monitoring of osteosarcoma, the pooled estimates included the sensitivity, specificity, positive likelihood ratio (PLR), negative likelihood ratio (NLR), diagnostic odd ratio (DOR) and the summary receiver operating characteristic curve (sROC) and AUC. Following recommendations of Cochrance Handbook (www.cochrance.org/trainig/cochrance-handbook), study-heterogeneity was evaluated using Chi-squared and I-square statistic algorithms. Low heterogeneity was defined as I-square < 50% and with P> 0.1. To obtain a reliable result, all analyses were performed using the random-effect model. The DOR is an indicator of the test accuracy, ranging from zero to infinity. A higher DOR indicates that the test is more accurate. The areas under the curve (AUC) and Q*-index are two important statistics that reflect the diagnostic value. The AUC is defined as the area under sROC, and Q* is the point where the sensitivity is equal to the specificity. The statistical analyses for the detection of primary lesions, recurrence, lung metastases, bone metastases and all distant metastases were performed separately.

## Results

### Study selection

**Fig. 1:**
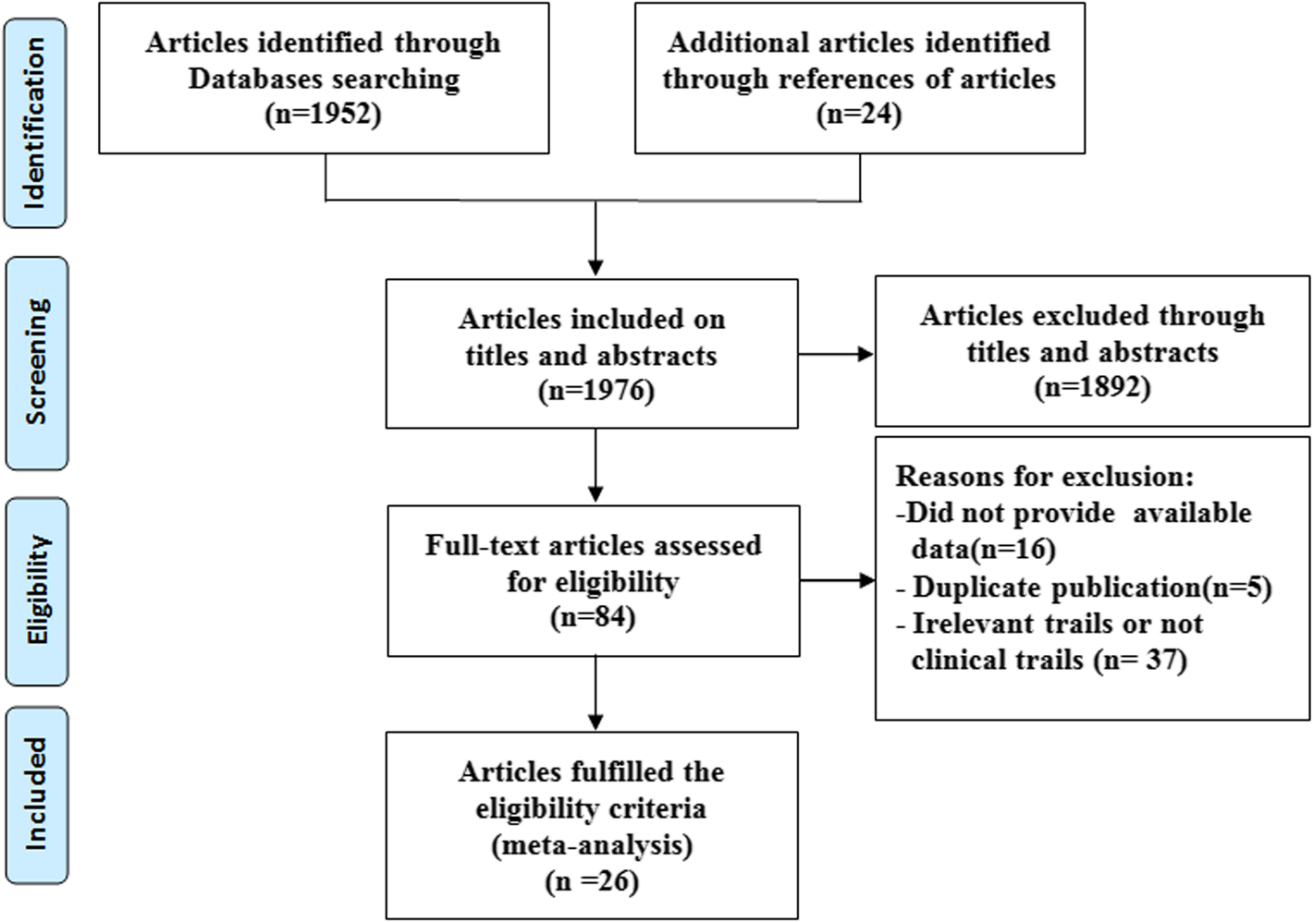
Selection process for studies included in the meta-analysis

At primary retrieval, a total of 1952 articles were identified, based on the search criteria, of which 1892 were subsequently deemed non-viable after inclusion and exclusion criteria were applied. Out of the 84 articles resulting from preliminary screening, 37 articles were referred to irrelevant studies, 5 articles were duplicate publications and 21 articles lacked sufficient data to calculate evaluation indicators and were excluded from the meta-analysis. The remaining 26 studies [13–38] deemed viable, published from 1998 to 2017, were included in the meta-analysis. Although five of the trials [14–18] showed signs of having utilized the same patients, these were included in a different subgroup analyses. The selection process and reasons other articles were excluded are described in Fig. 1.

### Characteristics of included studies

The basic characteristics of the 26 included studies are summarized in Table 1. The sizes of patients in the studies ranged from 5 to 206, and a total of 798 osteosarcoma patients were included. A total of 17 studies were retrospective design types whereas 8 studies were prospective. Based on the QUADAS score, only one study [22] had a score of 9, nine studies [14, 16–18, 25, 30, 31, 34, 35] had a score of 11, five studies [15, 21, 23, 24, 32] a score of 12 with all remaining studies [13, 19, 20, 26–29, 33, 36–38] possessing a score of 13. The major information of PET or PET/CT imaging of each study was summarized in Table 2. Among them, sixteen studies presented that image acquisition were performed approximately 60 min after FDG injection. The results of whole-body ^18^F-FDG PET or PET-CT were analyzed visually by experienced radiologists in 7 studies [13, 20, 22, 30, 31, 34, 37] and by standardized uptake values in 19 studies [14–19, 21, 23–29, 32, 33, 35, 36, 38]. The diagnostic data (TPs, FPs, TNs and FNs) were extracted directly or calculated through data provided in the tables or bodies of each original article (Table 3).

**Table 1:** Main characteristics of the included studies

Study, year	Country	No. of Subjects	Gender (M/F)	Median/Mean age (years)	Age range (years)	Design	Inclusion interval	QUADAS scores
Kole,1998 [13]	Netherlands	5	3/2	20/20.2	17–24	Retrospective	NR	13
Schulte,1999 [14]	Germany	27	17/10	17/NR	5–36	Prospective	Jan.1993-NR	11
Franzius,2000 [15]	Germany	32	NR	NR	NR	Retrospective	Aug.1995-Jun.1999	11
Schulte,2000 [16]	Germany	44	NR	NR	NR	Retrospective	Jan.1993-NR	12
Franzius,2001 [17]	Germany	32	NR	NR	NR	Retrospective	Aug.1995-Jun.1999	11
Franzius,2002 [18]	Germany	6	NR	NR	NR	Retrospective	NR	11
Yanagawa,2003 [19]	Japan	5	4/1	14/14.4	11–20	Prospective	Jun.1999-Mar.2000	13
Kneisl,2006 [20]	USA	38	NR	NR	NR	Retrospective	Dec.1994-Nov.2004	13
Iagaru,2006 [21]	USA	6	NR	NR	NR	Retrospective	Jan.2003-Dec.2005	12
Volker,2007 [22]	Germany	11	NR	NR	1–18	Prospective	Dec.2003-Oct.2006	9
Shin,2008 [23]	South Korea	7	NR	NR	6–79	Retrospective	May.2004-Jun.2007	12
Charest,2009 [24]	Canada	24	NR	NR	NR	Retrospective	May.2004-Apr.2008	12
Hawkins,2009 [25]	USA	40	NR	15.1/NR	7.1–31	Prospective	Jul.1995-Aug.2004	11
Benz,2010 [26]	USA	6	2/4	27/30.8	18–58	Prospective	Feb.2005-Nov.2007	13
Lindholm,2011 [27]	Finland	6	4/2	16.5/16.5	15–18	Prospective	NR	13
Bandopadhyaya,2012 [30]	India	22	14/8	21.55/NR	8–66	Prospective	NR	13
Cistaro,2012 [28]	Italy	11	NR	NR/14	NR	NR	NR	13
Ozkan,2012 [29]	Turkey	7	6/1	25/26.1	18–50	Retrospective	2007–2009	11
Bai,2013 [33]	China	14	9/5	NR/14.9	8–22	Retrospective	Jan.2009-Nov.2011	13
Byun,2013 [31]	South Korea	206	127/79	15/NR	4–71	Retrospective	Jan.2006-Nov.2011	11
Kong,2013 [32]	South Korea	26	16/10	NR/21	9–55	Prospective	May.2010-Aug.2011	12
Chang,2015 [35]	South Korea	109	74/35	NR/17	NR	Retrospective	Feb.2002-Sep.2012	11
Quartuccio,2015 [34]	Italy	20	10/10	15.5/NR	NR	Retrospective	Jan.2006-Sep.2010	11
Hurley, 2016 [36]	USA	39	19/20	Median 12	5–19	Retrospective	2003–2012	13
Angelini, 2017 [38]	Italy	37	20/17	Mean 20	7–52	Retrospective	2008–2014	13
Sharp, 2017 [37]	USA	8	5/3	Median 13	8–16	Retrospective	Oct.2004-Feb. 2013	13

*M* male, *F* female, *NR* not reported

**Table 2:** Technical aspects of the included studies

Study, year	Image device	Injected dose	Time between injection and image acquisition	PET image analysis
Kole, 1998 [13]	PET	370 MBq	50 min	Visual
Schulte, 1999 [14]	PET	120–300 MBq	45–60 min	Visual and semiquantitative
Franzius, 2000 [15]	PET	3.7 MBq/kg	60 min	Visual and semiquantitative
Schulte, 2000 [16]	PET	120–300 MBq	45–60 min	Visual and semiquantitative
Franzius, 2001 [17]	PET	3.7 MBq/kg	60 min	Visual and semiquantitative
Franzius, 2002 [18]	PET	3.7 MBq/kg	60 min	Visual and semiquantitative
Yanagawa, 2003 [19]	PET	4.5 MBq/kg	50 min	Visual and semiquantitative
Kneisl, 2006 [20]	PET	444–740 MBq	60 min	Visual
Iagaru, 2006 [21]	PET/CT	151.7–721.5 MBq	60 min	Visual and semiquantitative
Volker, 2007 [22]	PET	NR	NR	Visual
Shin, 2008 [23]	PET/CT	8.14 MBq/kg	60 min	Visual and semiquantitative
Charest, 2009 [24]	PET/CT	379–500 MBq	60 min	Visual and semiquantitative
Hawkins, 2009 [25]	PET	259–370 MBq	45 min	Visual and semiquantitative
Benz, 2010 [26]	PET/CT	7.77 MBq/kg	60 min	Visual and semiquantitative
Lindholm, 2011 [27]	PET/CT	370 MBq	60 min	Visual and semiquantitative
Bandopadhyaya, 2012 [30]	PET/CT	370 MBq	60 min	Visual
Cistaro, 2012 [28]	PET/CT	120–277 MBq	60 min	Visual and semiquantitative
Ozkan, 2012 [29]	PET/CT	555 MBq	60 min	Visual and semiquantitative
Bai, 2013 [33]	PET/CT	3.5–5.7 MBq/kg	40–60 min	Visual and semiquantitative
Byun, 2013 [31]	PET/CT	7.4 MBq/kg or 370 MBq	60 min	Visual
Kong, 2013 [32]	PET/CT	8.14 MBq/kg	NR	Visual and semiquantitative
Chang, 2015 [35]	PET/CT	7.4 MBq/kg or 370 MBq	60 min	Visual and semiquantitative
Quartuccio, 2015 [34]	PET/CT	113–596 MBq	72 min	Visual
Hurley, 2016 [36]	PET/CT	5.55 MBq/kg	60 min	Visual and semiquantitative
Angelini, 2017 [38]	PET/CT	5.55 MBq/kg	NR	Visual and semiquantitative
Sharp, 2017 [37]	PET/CT	5.18 or 5.55 MBq/kg	60 min	Visual

*NR* not reported

**Table 3:** Diagnosis accuracy data on each examination– or lesion–based analysis

Study, year	Total	TP	FP	FN	TN	Lesions sites
Kole, 1998 [13]	5	5	0	0	0	Primary lesion
Schulte, 2000 [14]	44	44	0	0	0	Primary lesion
Yanagawa, 2003 [19]	5	5	0	0	0	Primary lesion
Iagaru, 2006 [21]	6	6	0	0	0	Primary lesion
Volker, 2007 [22]	11	11	0	0	0	Primary lesion
Shin, 2008 [23]	7	7	0	0	0	Primary lesion
Charest, 2009 [24]	12	12	0	0	0	Primary lesion
Hawkins, 2009 [25]	40	40	0	0	0	Primary lesion
Benz, 2010 [26]	6	6	0	0	0	Primary lesion
Lindholm, 2011 [27]	6	6	0	0	0	Primary lesion
Bandopadhyaya, 2012 [30]	22	22	0	0	0	Primary lesion
Bai, 2013 [33]	14	14	0	0	0	Primary lesion
Kong, 2013 [32]	26	26	0	0	0	Primary lesion
Hurley, 2016 [36]	39	39	0	0	0	Primary lesion
Franzius, 2002 [18]	6	6	0	0	0	Recurrence
Charest, 2009 [24]	12	6	0	1	5	Recurrence
Cistaro, 2012 [28]	11	10	0	0	1	Recurrence
Ozkan, 2012 [29]	8	3	0	0	5	Recurrence
Chang, 2015 [35]	109	7	6	2	94	Recurrence
Angelini, 2017 [38]	37	22	0	1	14	Recurrence
Sharp, 2017 [37]	10	10	0	0	0	Recurrence
Schulte, 1999 [14]	27	4	0	0	23	Lung
Franzius, 2001 [17]	49	4	0	4	41	Lung
Bandopadhyaya, 2012 [30]	22	10	1	0	11	Lung
Cistaro, 2012 [28]	23	16	0	3	4	Lung
Ozkan, 2012 [29]	8	1	0	0	7	Lung
Bai, 2013 [33]	14	2	0	0	12	Lung
Quartuccio, 2015 [34]	56	27	5	5	19	Lung
Angelini, 2017 [38]	37	12	0	3	22	Lung
Volker, 2007 [22]	31	28	0	3	0	Bone
Ozkan, 2012 [29]	8	1	0	0	7	Bone
Bai, 2013 [33]	14	7	0	0	7	Bone
Byun, 2013 [31]	833	52	15	3	763	Bone
Quartuccio, 2015 [34]	21	16	1	0	4	Bone
Hurley, 2016 [36]	40	5	3	0	32	Bone
Schulte,1999 [14]	27	4	0	0	23	All metastatic lesions
Franzius, 2001 [17]	49	4	0	4	41	All metastatic lesions
Volker, 2007 [22]	42	31	0	3	8	All metastatic lesions
Bandopadhyaya, 2012 [30]	22	10	1	0	11	All metastatic lesions
Cistaro, 2012 [28]	38	27	2	3	6	All metastatic lesions
Ozkan, 2012 [29]	8	3	0	0	5	All metastatic lesions
Bai, 2013 [33]	28	9	0	0	19	All metastatic lesions
Byun, 2013 [31]	833	52	15	3	763	All metastatic lesions
Quartuccio, 2015 [34]	101	59	9	5	28	All metastatic lesions
Hurley, 2016 [36]	105	20	8	4	73	All metastatic lesions
Angelini, 2017 [38]	74	29	1	3	41	All metastatic lesions

*TP* True positive, *FP* False positive, *FN* False negative, *TN* True negative

### Primary lesion

The diagnosis of the primary lesion was analyzed on a patient-based level. Fourteen studies [13, 15, 19, 21–27, 30, 32, 33, 36] with 243 patients, with known osteosarcoma, were available. There were no threshold effects in this data (*p*-value = 1.000). All primary lesions were FDG-avid. The combined sensitivity of ^18^F-FDG PET and PET/CT in the detection of osteosarcoma was 100%, while no specificity could be obtained. There was no statistically significant heterogeneity in these estimates of sensitivity (I-square = 0.0%).

### Recurrence

**Fig. 2:**
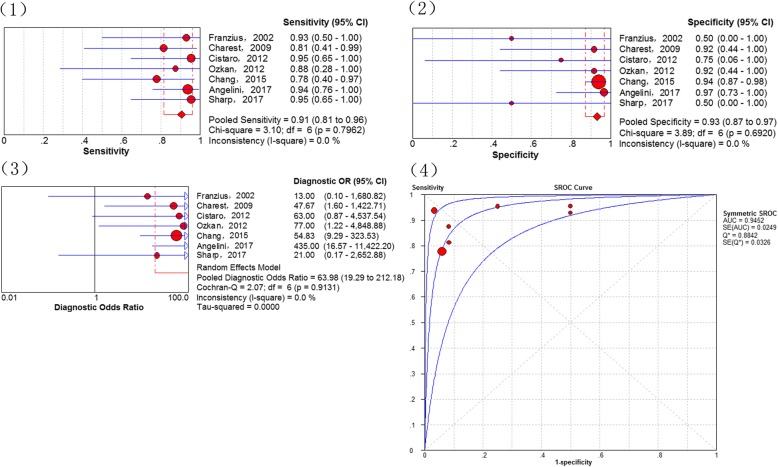
Diagnostic performance of 18F-FDG PET and PET/CT for the recurrence of osteosarcoma: (1) Pooled sensitivity (2) Pooled specificity (3) Pooled diagnostic odds ratio (4) Summary receiver operating characteristic curve (sROC) with the Q*-index

Results assessing the diagnostic performance of ^18^F-FDG PET and PET/CT for detecting recurrence of osteosarcoma as generated from the 7 studies [18, 24, 28, 29, 35, 37, 38], showed that the pooled sensitivity of 91% (95% CI of 81–96%), a specificity of 93% (95% CI of 87–97%), a PLR of 7.36 (95% CI of 3.54–15.30), a NLR of 0.14 (95% CI of 0.07–0.29), a DOR of 63.98 (95% CI of 19.29–212.18), the Q*-index of 0.8842 and AUC of 0.9452 (Fig. 2). No significance of heterogeneity between study for the pooled sensitivity (I-square = 0.0%) and the threshold effect (*p*-value = 0.269) were presented in this analysis.

### Lung metastases

**Fig. 3:**
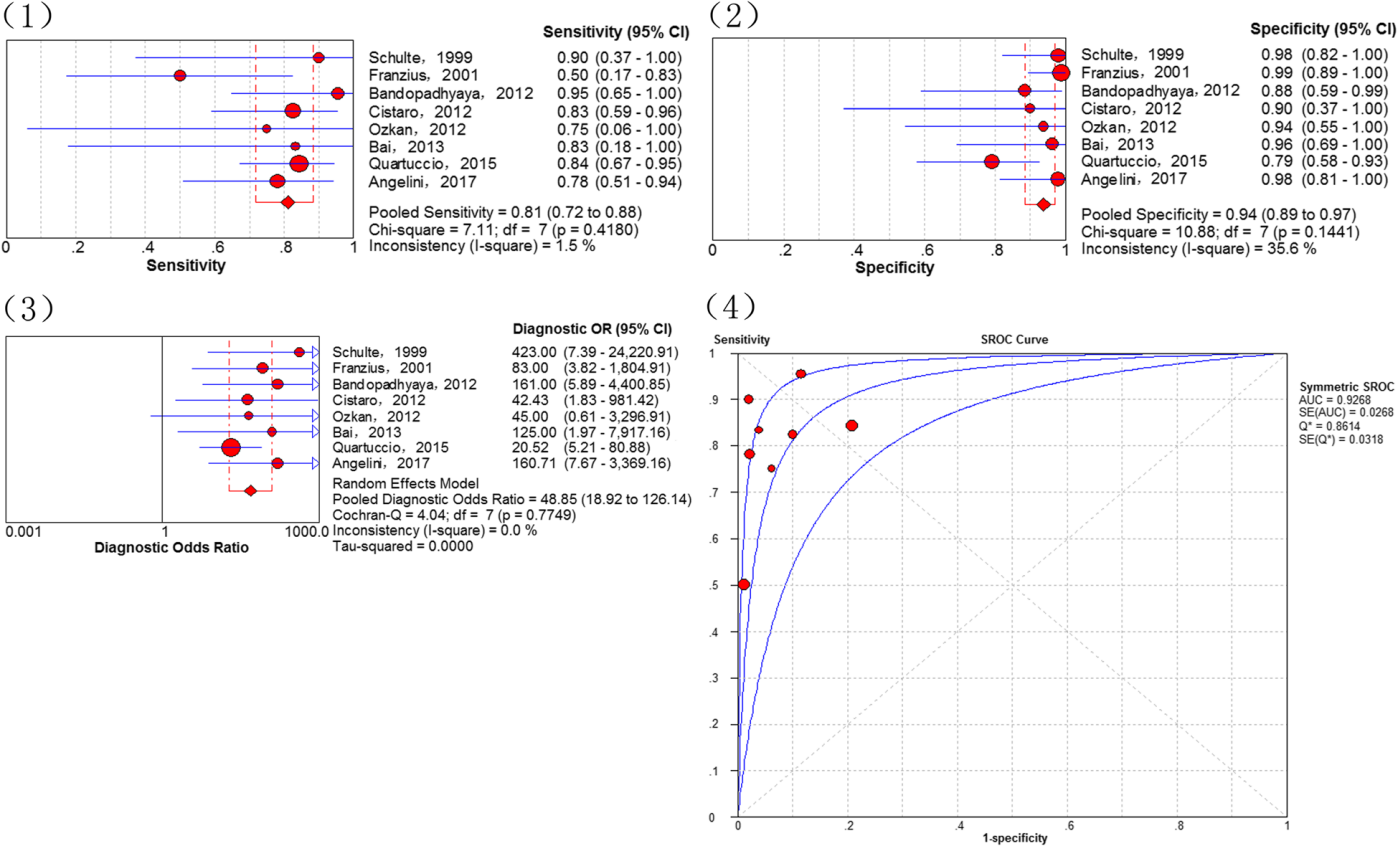
Diagnostic performance of 18F-FDG PET and PET/CT for osteosarcoma lung metastasis: (1) Pooled sensitivity (2) Pooled specificity (3) Pooled diagnostic odds ratio (4) Summary receiver operating characteristic curve (sROC) with the Q*-index

In total, 8 studies [14, 17, 28–30, 33, 34, 38] addressed the diagnosis of lung metastases. The threshold effects was no found in the data (p-value = 0.233). The combined sensitivity of ^18^F-FDG PET and PET/CT in detecting lung metastases of osteosarcoma was 81% (95% CI of 72–88%), specificity 94% (95% CI of 89–97%), PLR 8.13 (95% CI of 4.19–15.77) and NLR 0.26 (95% of CI 0.17–0.40). The pooled DOR was 48.85 (95% CI of 18.92–126.14), whereas the Q*-index was 0.8614 with an AUC of 0.9268 (Fig. 3).

### Bone metastases

**Fig. 4:**
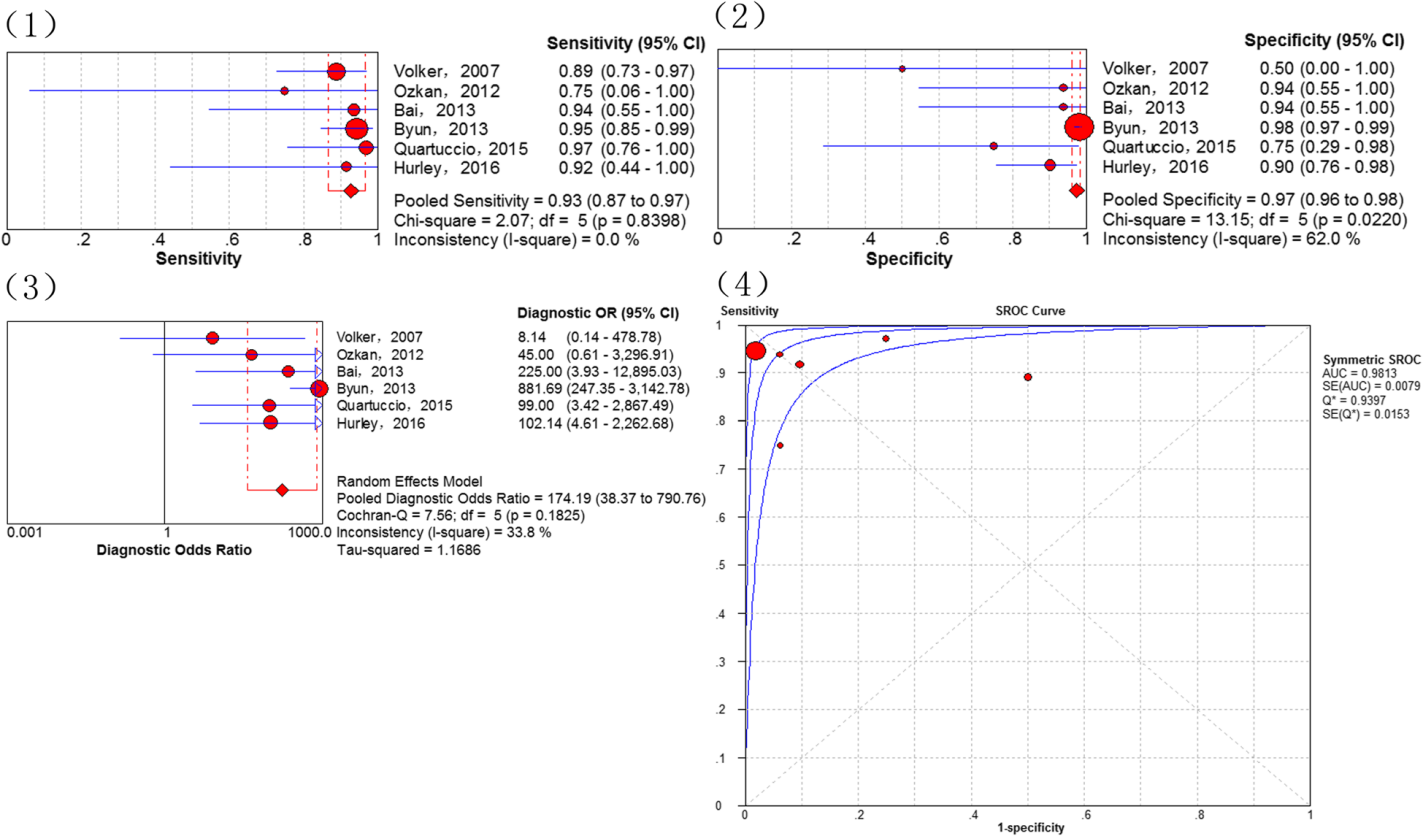
Diagnostic performance of 18F-FDG PET and PET/CT for osteosarcoma bone metastasis: (1) Pooled sensitivity (2) Pooled specificity (3) Pooled diagnostic odds ratio (4) Summary receiver operating characteristic curve (sROC) with the Q*-index

Results assessing the diagnostic performance of ^18^F-FDG PET and PET/CT for detecting bone metastasis of osteosarcoma as generated from the 6 studies [22, 29, 31, 33, 34, 36], demonstrated that the pooled sensitivity of 93% (95% CI of 87–97%), a specificity of 97% (95% CI of 96–98%), a PLR of 9.81 (95% CI of 2.73–35.29), a NLR of 0.08 (95% CI of 0.04–0.18), a DOR of 174.19 (95% CI of 38.37–790.76), the Q*-index of 0.9397 and the AUC of 0.9813 (Fig. 4). No significance of heterogeneity between study for the pooled sensitivity (I-square = 33.8%) and the threshold effect (*p*-value = 0.8279) were presented in this analysis.

### All distant metastases

**Fig. 5:**
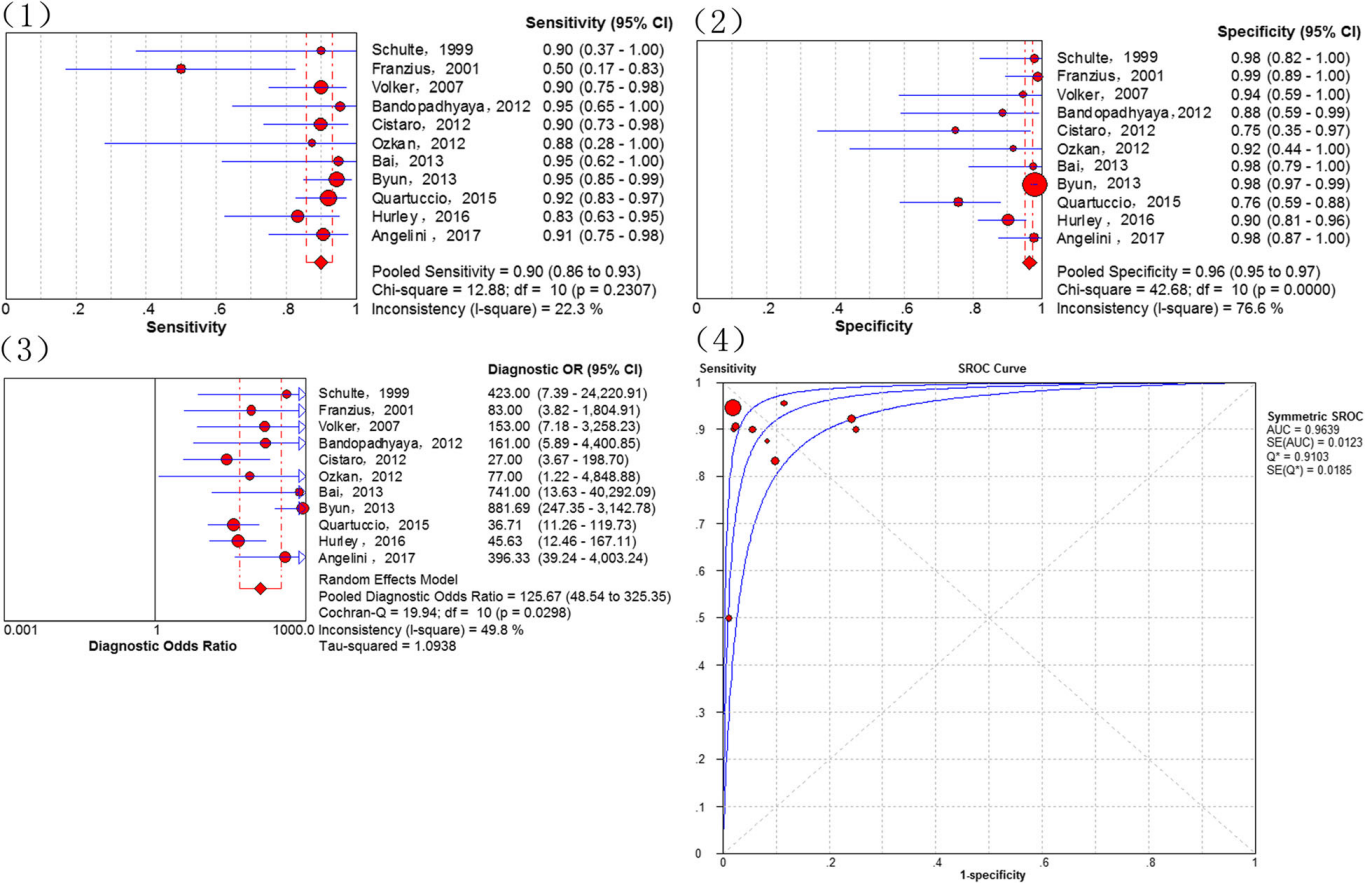
Diagnostic performance of 18F-FDG PET and PET/CT for all distant metastases of osteosarcoma: (1) Pooled sensitivity (2) Pooled specificity (3) Pooled diagnostic odds ratio (4) Summary receiver operating characteristic curve (sROC) with the Q*-index

In total, 11 studies [14, 17, 22, 28–31, 33, 34, 36, 38] addressed the diagnosis of all distant metastases. There were no threshold effects in this data (p-value = 0.647). The combined sensitivity of ^18^F-FDG PET and PET/CT in detecting all distant metastases of osteosarcoma was 90% (95% CI of 86–93%), specificity 96% (95% CI of 95–97%), PLR 13.81 (95% CI of 5.77–33.06) and NLR 0.13 (95% CI of 0.07–0.23). Statistically heterogeneity was no found in the estimates of the DOR (I-square = 49.8%). The pooled DOR was 125.67 (95% CI of 48.54–325.35). The Q*-index was 0.9103 with an AUC of 0.9639 (Fig. 5). To explore whether the injected dose of 18F-FDG could affect the diagnostic accuracy of all distant metastases, we performed a subgroup analysis which demostrated that 18F-FDG PET/CT, using a injected dose above 5 MBq/kg, is similar to that using a lower dose (sensitivity of 88% [95% CI 76–95%] vs. 88% [95% CI 78–95%]; specificity of 93% [95% CI 97–99%] vs. 98% [95% CI 97–99%]).

## Discussion

Osteogenic sarcoma has an elevated rate of glycolysis and, consequently, a high uptake of ^18^F-FDG in malignant cells [39] . A previous meta-analysis [40] revealed that the standardized uptake values before (SUV1) and after (SUV2) chemotherapy in osteosarcoma were associated with the histological response. SUV2:1< 0.5 or SUV2< 2.5 had predictive significance for tumor necrosis. Multiple studies investigated the diagnostic value of ^18^F-FDG PET and PET/CT for osteosarcoma, but no definitive results were obtained. To settle these questions scientifically, in the present study, a rigorous inclusion criterion was predesigned to collect published articles as comprehensive as possible and subgroup analyses were conducted to pool outcome estimates from individual studies only utilizing the random-effect modeling. Importantly, this is the first meta-analysis evaluating the diagnostic utility of ^18^F-FDG PET/CT in osteosarcoma. By systemically analyzing the retrieved data, investigations demonstrated that ^18^F-FDG PET/CT had an excellent accuracy in the diagnosis, staging and restaging of osteosarcoma.

The value of PET in the differential diagnosis of bone tumor and tumor-like lesions was first described by Schulte et al.(2000) [15]. Using a tumor-to-background ratio of 3.0 as the cutoff for determining malignant bone lesions, the sensitivity, specificity and accuracy were 0.93, 0.667 and 0.817, respectively. Although there were several false negative cases, there were none for patients with osteosarcoma (*n* = 44). A subsequent meta-analysis [41] also reported the outstanding ability of ^18^F-FDG PET to distinguish between benign and malignant bone and soft tissue tumors. In studies included in the present meta-analysis, all primary osteosarcoma lesions (*n* = 243) were ^18^F-FDG-avid with a good pooled sensitivity of 100%. However, it must be noted, that as a nonspecific manifestation on ^18^F-FDG PET, osteosarcoma is not distinctly discernible from other highly malignant bone sarcomas, such as Ewing sarcoma [42].

Detecting recurrent or residual osteosarcoma has been a challenge for clinicians due to the post-therapeutic changes and imaging artifacts caused by metallic endoprothesis [43]. In 1996, Garcia and co-workers reported the diagnostic value of ^18^F-FDG PET in 48 suspected recurrent musculoskeletal sarcomas (including 18 osteosarcoma patients), indicating a good sensitivity and specificity (98 and 90%, respectively) [44]. ^18^F-FDG PET has an advantage in assessing local recurrence because it is not affected by the imaging artifact. Nevertheless, elevated ^18^F-FDG uptake could be affected by post-treatment changes, irrespective of local recurrence [45, 46]. In this investigation, a good accuracy was observed of ^18^F-FDG PET in detecting recurrent osteosarcoma (local relapses or distant metastases), which are similar to the conclusions for other recurrent malignant tumors [47, 48].

Although osteosarcoma has a tendency to metastasize early, which would modify the outcome of osteosarcoma with an unfavorable survival, the prognosis of patients with resectable metastatic lesions is relatively good [49–51]. ^18^F-FDG PET and PET/CT are useful tools for detecting possible malignant lesions. Therefore, a subgroup analysis was performed on the metastasis-based data in the presented study.

Lung metastasis ranks as the leading cause of osteosarcoma-related death [2]. Schulte et al. (1999) [14] first reported the diagnostic performance of PET for detecting osteosarcoma lung metastasis. In the presented investigation a total of 27 scans included 4 true positive and 23 true negative cases. However, Iagaru et al. (2006) [20] of 106 bone and soft tissue sarcomas suggested that PET-false positive cases were significantly increased in sub-centimeter lung metastases. Cistaro et al. (2012) [28] published data on 18 participants (11 osteosarcomas), who underwent 31 PET/CT scans where it was demonstrated that there was no significant advantage of the SUVmax or SUVratio in the evaluation of lung nodules of <6 mm. This may explain the lower sensitivity of ^18^F-FDG PET and PET/CT in the diagnosis of lung metastases compared to bone metastases in the present meta-analysis. Compared to those who only have lung metastasis, the survival for patients with osteosarcoma who have bone metastasis is even poorer [5]. Because there are many possible metastatic bones in osteosarcoma, whole-body screening is indispensable. In the diagnosis of bone metastases, our investigations revealed a remarkable sensitivity, specificity and Q*-index (0.93, 0.97 and 0.9397, respectively) for ^18^F-FDG PET and PET/CT. Bone scintigraphy is another commonly used functional modality, while previous studies have shown that ^18^F-FDG PET and PET/CT are superior to bone scintigraphy in detecting bone metastases of osseous sarcoma, including osteosarcoma [34]. Other than metastasis in the lungs and bones, osteosarcoma could metastasize to other locations such as the lymph nodes and soft tissue. Because these cases are rare, only distant metastases as a whole were analyzed. The present analysis further demonstrated a diagnostic accuracy of ^18^F-FDG uptake in metastatic lesions with an AUC of 0.9639 and *Q of 0.9103.

Misdiagnoses of ^18^F-FDG PET and PET/CT are attributable to multiple reasons. Three included studies [34–36] showed higher FN and FP than the others because one study [35] detected local recurrence in patients with extremity osteosarcoma treated with surgical resection and endoprosthetic replacement and another two studies [34, 36] involved newly diagnosed with high grade osteosarcoma or initial staging osteosarcoma patients. Increased ^18^F-FDG uptake is not pathognomonic for malignancy. Some benign lesions, such as giant cell tumors of the bones, inflammatory disease and fractures are presented with a high level of tracer accumulation [6]. Among these, inflammatory disease is the most commonly encountered causes of a false positive. Therefore, the findings of ^18^F-FDG PET/CT for malignant lesions should be confirmed with a histopathology examination or follow-up. Meanwhile, some false negative cases are unavoidable. One cause is the nonspecific ^18^F-FDG uptake in malignant diseases and asymmetric ^18^F-FDG distribution. Second, because of the limited spatial resolution of ^18^F-FDG PET, some occult or sub-centimeter lesions could not be identified. Currently, the high cost of ^18^F-FDG PET and PET/CT discourages their use in developing countries [52–54]. However, considering their satisfactory performance, as demonstrated by the present investigation, and the poor prognosis of osteosarcoma, especially for those with recurrence or distant metastases, patients may benefit from evaluation with ^18^F-FDG PET or PET/CT.

Some limitations of this meta-analysis merit considerations. First, the present analysis was a meta-analysis and systemic review; therefore, we were only able to analyze questions on the study level instead of on the patient level. Second, as a result of a lack of publications assessing the effectiveness of ^18^F-FDG PET and PET/CT for detecting osteosarcoma, several sub-group analyses in our investigation were performed on a small number of studies. Third, there was methodological variability in the included studies, such as in the SUV, reference standards tests and duration of follow-up. Furthermore, some studies were assessed using ^18^F-FDG PET, while others were assessed with PET/CT. Finally, some information was not available in the included studies.

## Conclusions

In summary, our comprehensive meta-analysis of publications demonstrated that ^18^F-FDG PET/CT have an excellent accuracy in the diagnosis, staging and recurrence monitoring of osteosarcoma. ^18^F-FDG-avid lesions should be further examined in osteosarcoma, especially for suspicious lung lesions. To support the current conclusions, larger-scale trials should now be conducted.

## Abbreviations

^18^F-FDG: 18F-fluorodeoxyglucose
AUC: Area under the curve
CI: Confidence interval
CT: Computed tomography
DOR: Diagnostic odd ratio
FN: False-negative
FP: False-positive
MRI: Magnetic resonance imaging
NLR: Negative likelihood ratio
PET: Positron emission tomography
PLR: Positive likelihood ratio
PRISMA: Preferred Reporting Items for Systematic Reviews and Meta-Analyses
sROC: The summary receiver operating characteristic curve
SUV: Standardized uptake value
TN: True negative
TP: True-positive
